# Identification of pregnancy-associated plasma protein A as a migration-promoting gene in malignant pleural mesothelioma cells: a potential therapeutic target

**DOI:** 10.18632/oncotarget.1126

**Published:** 2013-07-08

**Authors:** Jun Huang, Sho Tabata, Soji Kakiuchi, Trung The Van, Hisatsugu Goto, Masaki Hanibuchi, Yasuhiko Nishioka

**Affiliations:** ^1^ Department of Respiratory Medicine and Rheumatology, Institute of Health Biosciences, The University of Tokushima Graduate School, Tokushima, Japan

**Keywords:** malignant pleural mesothelioma, pregnancy-associated plasma protein A, migration, orthotopic xenograft mouse model

## Abstract

Despite recent advances in treatment, malignant pleural mesothelioma (MPM) remains a deadly disease. Targeted therapy generated broad interests and is highly expected for the treatment of MPM, yet promising preclinical results have not been translated into substantial clinical benefits for the patients. In this study, we tried to identify the genes which play functional roles in cell migration as well as to test whether they can be used as novel targets for molecular targeted therapy for MPM in preclinical model. In our study, pregnancy-associated plasma protein A (*PAPPA*) was identified as a gene whose expression level is correlated with MPM cell migration by correlation analysis combining MPM cell migration ability and their gene expression profiles. Highly migratory cells were selected from MPM cell lines, MSTO-211H, NCI-H290 and EHMES-1 *in vitro* and up-regulation of *PAPPA* in these cells were confirmed. *In vitro*, PAPPA was demonstrated to stimulate the MPM cell migration via cleavage of insulin-like growth factor-binding protein-4 and subsequent release of IGF-1. Gene silencing of *PAPPA* in MPM cells led to reduced migration, invasion and proliferation. Furthermore, *PAPPA* shRNA transfected NCI-H290 when orthotopically inoculated into pleural cavity of severe combined immunodeficiency recipient mice, failed to develop tumors and produce bloody pleural effusion as control shRNA transfected cells did. Our study suggests that *PAPPA* plays a functional role in promoting MPM cell migration and it might serve as a potential therapeutic target for the treatment of MPM.

## INTRODUCTION

Malignant pleural mesothelioma (MPM), the most common mesothelioma, is an aggressive tumor associated with prior asbestos exposures that arise from mesothelial surface of pleura [1]. Despite recent advances in treatment, MPM remains a deadly disease. The benefit from surgery is confined to a minority of patients. In addition, cytotoxic chemotherapeutic drugs improve quality of life for unresectable patients, and can prolong survival, though only by a few months at best [2]. There has been substantial interest in the role of targeted agents in the treatment of MPM, a variety of them has been tested as therapy for this malignancy in preclinical studies [3-5], yet promising preclinical data has not been translated into clinical benefits in a significant way [6]. More about the biology of this disease might be needed for the molecular targeted therapy to yield promising new treatment options.

Even though metastases are rarely the cause of death of MPM patients, the unique clinical feature of local spread along tissue planes not only causes increasing pain and functional abnormalities, but also makes it a difficult neoplasm to manage [1, 7]. Cell migration as a key to local invasion, might provide an opening for therapeutic approaches if we can understand more about the molecular biology that underlie it [8]. The goal of our study was to identify the genes which might be involved in the regulation of cell migration as well as to evaluate whether they can be used as targets for the inhibition of MPM cell migration, invasion or even tumor progression.

To achieve this goal, we investigated the migration ability of 8 human MPM cell lines *in vitro* and their gene expression profiles. Genes whose expressions correlated with cell migration were identified by correlation analysis combining the migration ability of MPM cells and their gene expression profiles. Among these genes, pregnancy-associated plasma protein A (*PAPPA*) caught our attention. *PAPPA*, one of four proteins of placental origin found circulating at high concentrations in pregnant women [9], is now basically recognized as a local regulator of Insulin-like growth factor 1 (IGF-1) bioavailability through cleavage of IGF-binding proteins (IGFBPs) [10-12]. The IGF axis was reported to be involved in the pathogenesis of many cancer types [13], including MPM [14-16], and therapeutics aimed at the IGF axis are under extensive clinical investigation [17]. Accumulating preclinical evidences support the idea that *PAPPA* may play a role in cancer [18-22].

In the present study, a series of *in vitro* assay was performed to try to reveal the mechanism of the migration-promoting role of *PAPPA*. To further confirm the causal role of *PAPPA* in MPM cell migration, we applied both small interfering RNA (siRNA) and small hairpin RNA (shRNA) targeting *PAPPA* to investigate the silencing effects of *PAPPA* on MPM cell migration. Finally, we used an orthotopic xenograft mouse model of MPM [3-5, 24] to evaluate the efficacy of *PAPPA* silencing on tumor development and progression *in vivo*.

## RESULTS

### Expression of PAPPA is correlated with migration ability in MPM cell lines

**Figure 1:**
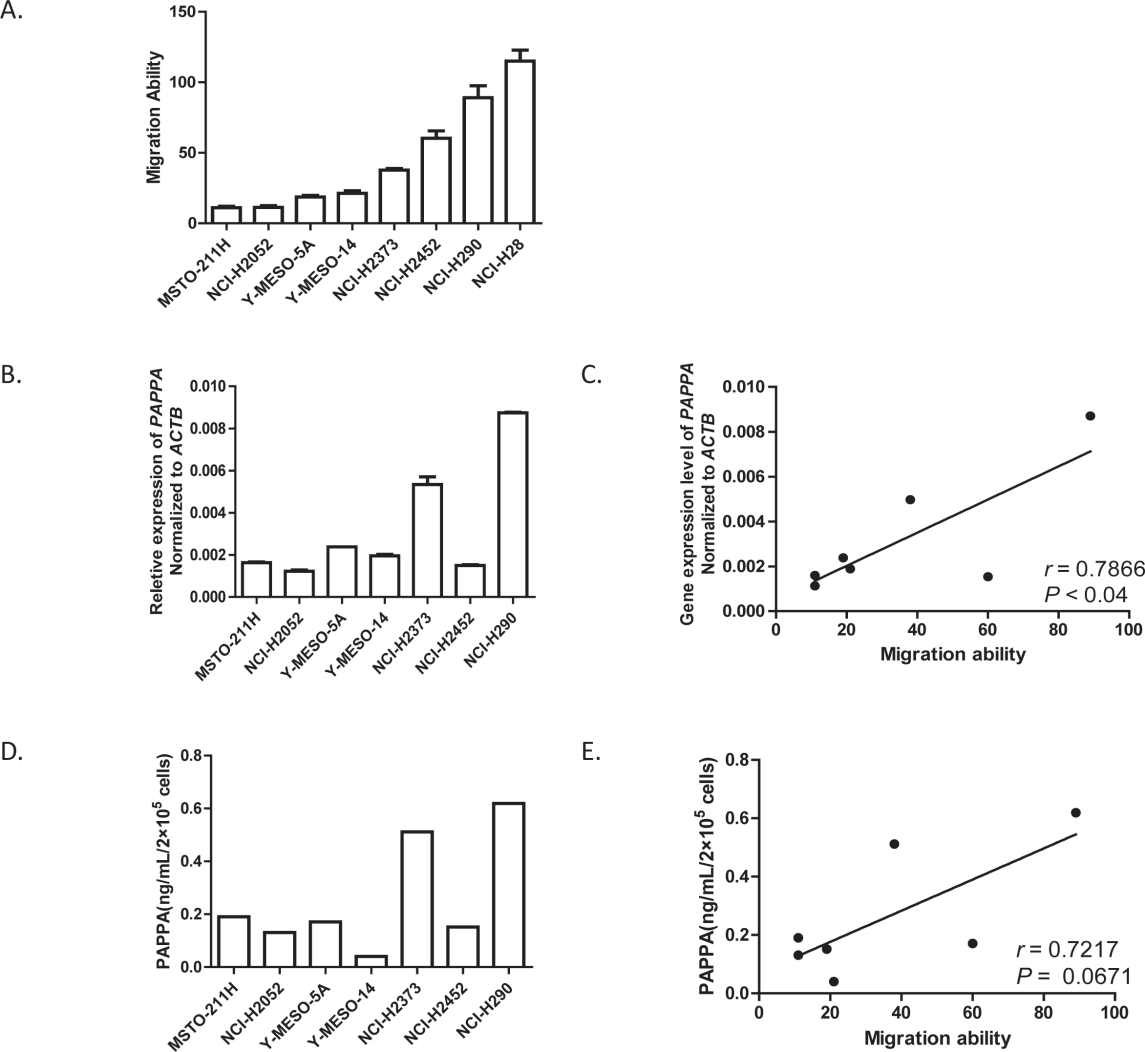
Migration abilities of malignant pleural mesothelioma cells are positively correlated with their expression levels of PAPPA A, The migration abilities of eight MPM cell lines determined by transwell migration assay, data are the means ± SEMs of three independent experiments. B, The expression levels of *PAPPA* in eight MPM cell lines determined by qRT-PCR. Data are representative of two independent assays carried out in triplicate (means ± SDs, n=3). C, The expression levels of *PAPPA* in eight MPM cell lines determined by qRT-PCR, were positively correlated with their migration ability. * *P*<0.05. The correlation efficient (r) and the significance (*P*) were calculated by Pearson's correlation analysis. D, The concentrations of secreted PAPPA in the conditioned medium (48 h) were determined by ELISA. E, The concentrations of secreted PAPPA from MPM cells (D) tend to have positive correlation with their migration abilities. *P*=0.0671. The correlation efficient (*r*) and the significance (*P*) were calculated by Pearson's correlation analysis.

To identify potential candidate genes whose function are related with MPM cell migration, we determined the migration ability of eight MPM cell lines using transwell chamber migration assay (Fig. 1A), and obtained their gene expression profiles using Affymetrix whole-genome oligonucleotide microarray (Supplementary Fig. 1A). After we applied Pearson's correlation by combining the migration ability of MPM cell lines and their gene expression profiles, expression levels of several genes revealed relatively high correlations with MPM cell migration ability (Supplementary Fig. 1B). Among these genes, we focused on *PAPPA*, which encodes a secreted metalloproteinase that degrades inhibitory IGFBPs. We validated our microarray expression data for *PAPPA* with quantitative real time PCR (qRT-PCR) (Fig. 1B). Overall, *PAPPA* expression results generated from the two different techniques were well correlated (Supplementary Fig. 1B), except that of NCI-H28. Thus we did not include NCI-H28 in the following studies. The similarity of expression levels of *PAPPA* obtained from microarray and qRT-PCR can also be extended to their correlations with MPM cell migration ability (Supplementary Fig. 1B, Fig. 1C). Considering that PAPPA functions as a secreted protein, we further determined the secreted protein levels of PAPPA in the conditioned medium (48 h) of MPM cell culture (Fig. 1D). The secreted PAPPA levels of MPM cell lines are also tend to be positively correlated with their migration ability (Fig. 1E, Pearson r = 0.7217, *P* = 0.0671).

**Figure 2:**
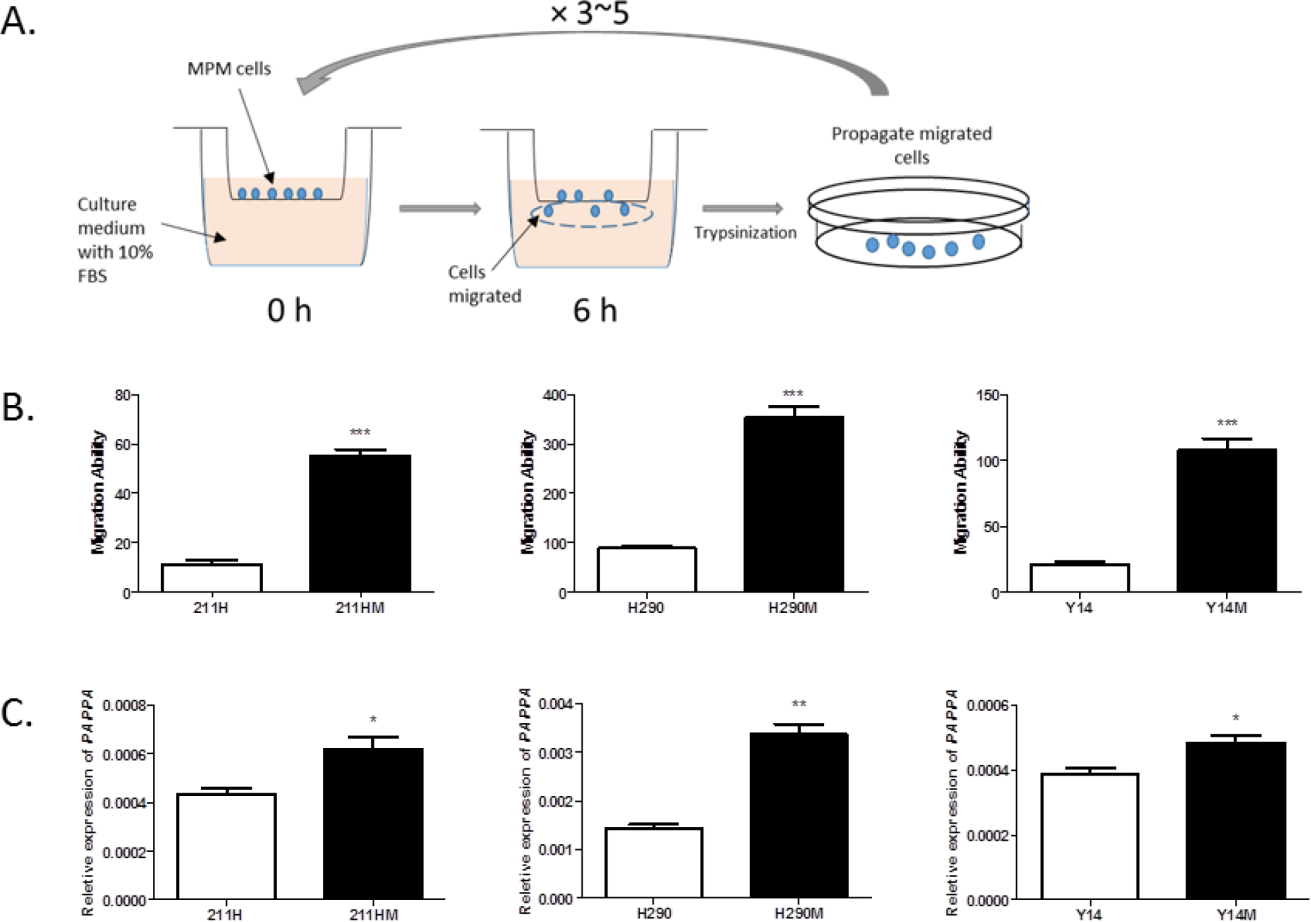
Selected highly migratory MPM cells and their PAPPA expression levels A, Schematic representation of *in vitro* selection of highly migratory cells. B, The cells that went through *in vitro* selection process revealed higher migration ability (means ± SDs, n=3). Left, NCI-211H parental cell (211H) versus NCI-211 highly migratory cells (211HM). *** *P*<0.001, unpaired *t* test; middle, NCI-H290 parental cell (H290) versus NCI-H290 highly migratory cells (H290M). *** *P*<0.001, unpaired *t* test; right, Y-MESO-14 parental cells (Y14) versus Y-MESO-14 highly migratory cells (Y14M). *** *P*<0.001, unpaired *t* test. C, The expression levels of *PAPPA* in selected cells were significantly higher as compared with that in parental cells. The data are representative of two independent assays carried out in triplicate (means ± SDs, n=3). Left, 211H versus 211HM. * *P*<0.05, unpaired *t* test; middle, H290 versus H290M, * *P*<0.05, unpaired *t* test; right, Y14 versus Y14M, * *P*<0.05, unpaired *t* test.

Considering the possibility that the correlation is simply due to coincidence, rather than the functional role of *PAPPA* in the regulation of MPM cell migration, we applied an *in vitro* selection strategy to further examine this correlation (Fig. 2A). Highly migratory MPM cells were selected, and their gene expression levels were examined. Three MPM cell lines, MSTO-211H, NCI-H290 and Y-MESO-14, after 3-5 round *in vitro* selection, increased their migration ability roughly 5-fold compared to their corresponding parental cell lines (Fig. 2B). Interestingly, when analyzing their gene expression profiles with microarray, we found that the genes including *PAPPA*, which we previously identified whose expression levels were positively correlated with migration ability (Supplementary Fig. 1B), were also expressed moderately higher in selected highly migratory cells as compared with their parental cell lines (Supplementary Table 1, Supplementary Fig. 3). Increased expression of *PAPPA* in highly migratory cells was further validated by qRT-PCR (Fig. 2C). These observations strongly indicated that there is an inherent relationship between *PAPPA* expression and migration ability in MPM cells.

### Major components of the IGF system are expressed in MPM cell lines

**Figure 3:**
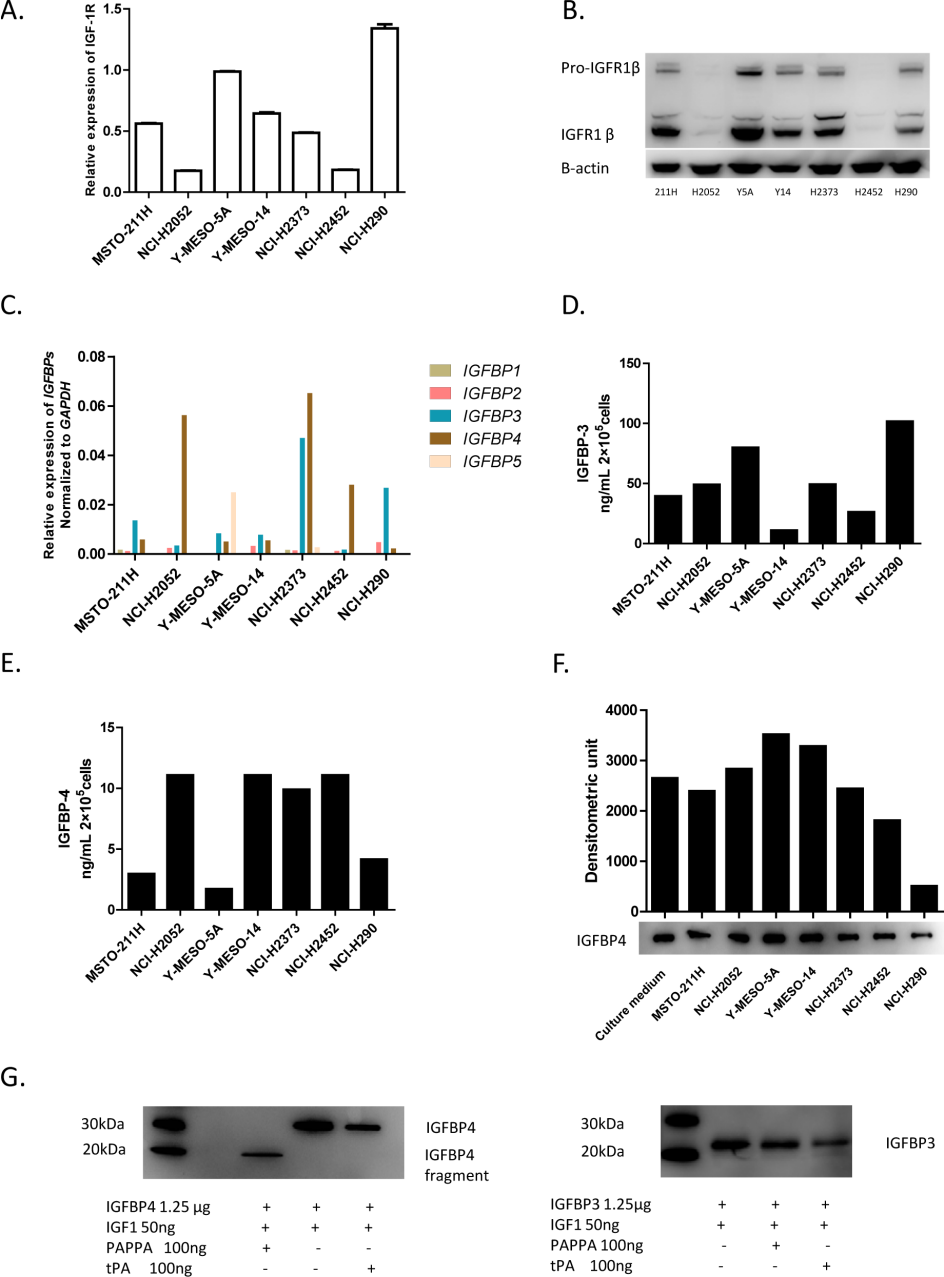
Characterization of major IGF components expression in MPM cell lines A, Relative gene expression levels of IGF-1R in MPM cell lines assessed by qRT-PCR, normalized to the relative expression of IGF-1R in Y-MESO-5A cells. The data are representative of two independent assays carried out in triplicate (means ± SDs, n=3). B, IGF-1R1 expression detected by immunoblotting. C, Relative gene expression levels of *IGFBP*s in MPM cell lines characterized by qRT-PCR, normalized to that of *ACTB*. D, The concentrations of secreted IGFBP-3 in the conditioned medium (24 h) of MPM cells detected by ELISA. E, The concentrations of secreted IGFBP-4 in the conditioned medium (24 h) of MPM cells by ELISA. F, The conditioned medium of MPM cell lines revealed enzymatic activity of cleaving IGFBP-4. For the detection of IGFBP-4 proteolysis, rhIGFBP-4 (0.5 μg), rhIGF-1 (100 ng) were add into 50 μl conditioned medium of MPM cells and incubated for overnight at 37°C, then conditioned medium was subjected to immunoblotting analysis. G, The enzymatic activity of conditioned medium of mesothelioma cell lines. Left panel, rhIGF-1, rhIGFPB-4 was incubated with or without rhPAPPA for 24 h, and then subjected to immunoblotting analysis. rhPAPPA but not rhtPA revealed specific enzymatic activity of cleavage of IGFBP4. Right panel, rhtPA specifically cleaved rhIGFBP-3.

PAPPA has long been identified as a local regulator of IGF bioavailability through cleavage of IGFBP4 [10-12], while IGF-1 has already been reported that it can induce MPM cell migration, a biological effect associated with IRS-2 phosphorylation [15]. We speculated that *PAPPA* may play a functional role in regulation of migration of MPM cells, at least partially, through the IGFBP-4/IGF-1/IGF-1R1 axis. Thus we characterized the major components of the IGF axis in MPM cell lines we used. IGF-1R expressions were detected at both mRNA and protein levels (Fig. 3A, 3B), which is consistent with previous report [23]. For IGF-1, no significant levels of secreted IGF-1 were detected by ELISA, and actually no mRNA of *IGF1* was detected by qRT-PCR (data not shown). For IGFBPs, we examined the mRNA transcript profiles of *IGFBP1* to *5* of MPM cells, notably, *IGFBP3* and *IGFBP4* were found to be the dominant IGF-1 binding proteins (Fig. 3C) expressed in MPM cell lines. We subsequently measured the concentrations of secreted IGFBP-3 (Fig. 3D) and IGFBP-4 (Fig. 3E) in the conditioned medium of MPM cell cultures. To test whether the PAPPA detected in the MPM cell conditioned medium is enzymatically active, we incubated rhIGFBP-4, rhIGF-1 with conditioned medium for 24 h, and subjected the conditioned medium to immunoblotting analysis. The conditioned medium of MPM cells did reveal enzymatic activity, as indicated by reduced rhIGFBP-4 after incubation (Fig. 3F). And the enzymatic activity of the conditioned medium seems to be correlated with the concentrations of PAPPA in these medium (Fig. 3F, Fig. 1D). Since tissue plasminogen activator (tPA) was reported to have IGFBP-3 proteolytic activity [24], and we also detected that PLAT (gene encoding tPA protein) is expressed in MPM cell lines (Supplementary Fig. 1B), we performed an *in vitro* proteolysis analysis to see whether the enzymatic cleavage of IGFBP-4 of conditioned medium of MPM cells can also be attributed to tPA. It turned out that PAPPA revealed proteolytic activity of IGFBP-4 but not IGFBP-3, while tPA revealed proteolytic activity of IGFBP-3 but not IGFBP-4 (Fig. 3G), supporting the idea that PAPPA is specifically responsible for the IGFBP-4 proleolytic activity of MPM cell conditioned medium. Taken together, the major components of the IGF axis are expressed in MPM cells, which gives the context of functional role of PAPPA as local regulator of IGF-1 for MPM cells.

### PAPPA enhanced migration of MPM cells by enzymatically cleaving inhibitive IGFBP-4 and releasing IGF-1 as chemotactic factor

**Figure 4:**
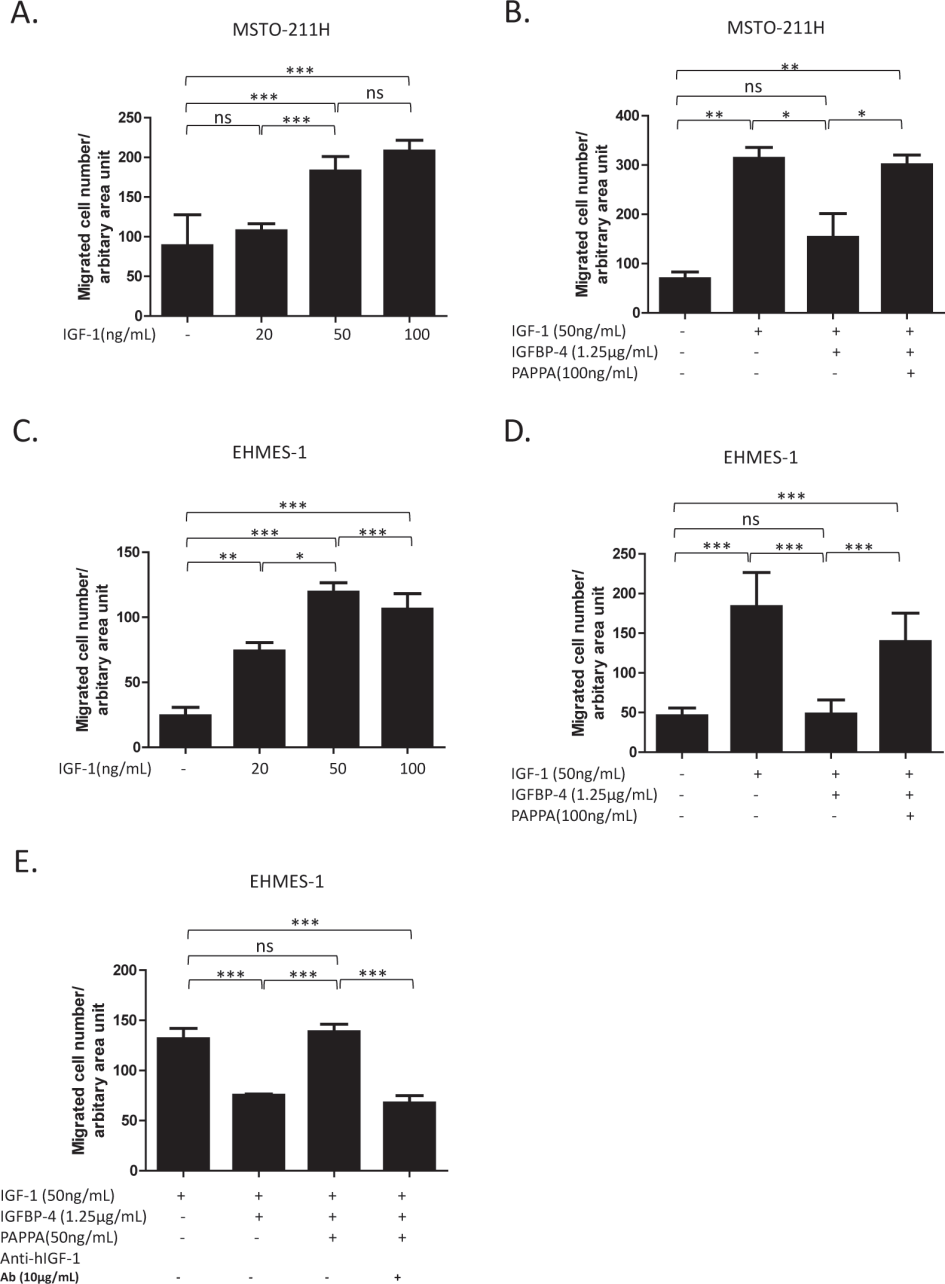
PAPPA enhances migration of MPM cells by enzymatically cleaving inhibitive IGFBP-4 and releasing IGF-1 as chemotactic factor A, rhIGF-1 induced migration of MSTO-211H cells in a dose-dependent manner. B, rhIGFBP-4 inhibited migration of MSTO-211H cells induced by rhIGF-1, while rhPAPPA recovered the ability of rhIGF-1 to induce migration of MSTO-211H. rhIGF-1 or rhIGF-1 + rhIGFBP-4 or rhIGF-1 + rhIGFBP4 + rhPAPPA were added in serum free medium and incubated at 37°C for 6 h before added into the lower chamber. C, rhIGF-1 attracted EHMES-1 cells to migrate dose-dependently. D, rhPAPPA recovered the ability of rhIGF-1 to induce the migration of EHMES-1 which was inhibited by rhIGFBP-4. E, An anti-IGF-1 antibody blocked the reversing effect of rhPAPPA on the migration of EHMES-1. Six-hours after the incubation with the rhIGF-1, rhIGFBP-4 and rhPAPPA mixture, an anti-IGF-1 antibody was added into the mixture and incubated for another 30 min before being added into the lower chambers. The data are representative of at least two independent assays carried out in triplicate (means ± SDs, n=3).* *P*<0.05, ** *P*<0.01, *** *P*<0.001, ns=not significant, one way ANOVA analysis.

To test the hypothesis that PAPPA could affect MPM cell migration via IGFBP-4/IGF-1/IGF-1R axis, a series of experiments were performed using transwell chamber assays with related recombinant proteins. Consistent with previous reports [15, 25], IGF-1 alone could stimulate the migration of EHMES-1 and MSTO-211H cells across a porous membrane in a dose-dependent manner (Fig. 4A, Fig. 4C). We also demonstrated that stimulating effect of IGF-1 could be blocked by IGFBP-4, while further addition of PAPPA would recover the stimulating effects of IGF-1 on MPM cell migration (Fig. 4B, Fig. 4D). PAPPA alone added to the lower chamber has no effects on cell migration (data not shown), excluding the possibility that the enhanced migration of MPM was due to the direct chemotactic effect of PAPPA. To further confirm that the released IGF-1, rather than the fragments of IGFBP-4 generated by cleavage, recovered the enhanced cell migration, anti-IGF-1 antibody was used to neutralize the released IGF-1 from IGFBP-4 proteolysis caused by PAPPA. The enhanced cell migration was completely abrogated by anti-IGF-1 antibody (Fig. 4E). Taken together, our results suggest that PAPPA could enhance the migration of MPM cell via proteolysis of IGFBP-4 and subsequently releasing IGF-1 as chemotactic factor.

### Silencing PAPPA by small interfering RNA inhibited migration and proliferation of MPM cells

**Figure 5:**
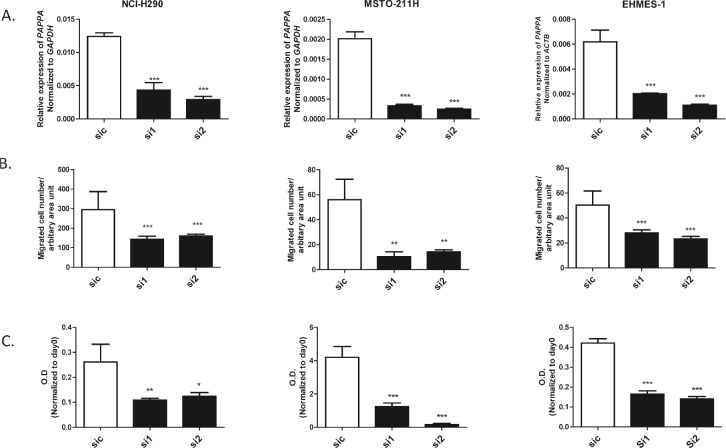
Effects of knockdown of PAPPA by siRNA on migration and proliferation of MPM cells A, The expression of *PAPPA* in MPM cells (left, NCI-H290; middle, MSTO-211H; right, EHMES-1) was analyzed by qRT-PCR 24 h after transfection with siRNA targeting *PAPPA*. B, The migration ability of MPM cells (left, NCI-H290; middle, MSTO-211H; right, EHMES-1) were determined by transwell migration assay 24 h after transfection with siRNA targeting *PAPPA*. C, The proliferation of MPM cells (left, NCI-H290; middle, MSTO-211H; right, EHMES-1) was determined by MTT assay 72 h after transfection with siRNA targeting *PAPPA*. The data are representative of two independent assays carried out in triplicate (means ± SDs, n=3), * *P*<0.05, ** *P*<0.01, *** *P*<0.001, one way ANOVA analysis.

To investigate the function of endogenous *PAPPA* on MPM cell migration more directly, we knocked down *PAPPA* in MPM cells (NCI-H290, MSTO-211H and EHMES-1) using siRNA and subsequently evaluated their migration ability. Efficacy of *PAPPA* silencing by siRNA were confirmed by qRT-PCR before the following functional assays (Fig. 5A). We found that *PAPPA* silencing significantly reduced cell migration (Fig. 5B). No difference of cell viability was observed at the time point of migration (24-30 h after siRNA treatment), as revealed by MTT assay (data not shown), but the inhibition of cell growth was also observed later, which became evident 72 h after *PAPPA* siRNA treatment (Fig. 5C). These data confirmed the functional roles of *PAPPA* on the migration of MPM cells and revealed an extended role of *PAPPA* on the proliferation of these cells, indicating it might be directly used as therapeutic target to inhibit progression of MPM *in vivo*.

### Transfection of PAPPA shRNA inhibited migration, invasion and proliferation of NCI-H290 cell line in vitro and tumor growth in orthotopic xenograft model

**Figure 6:**
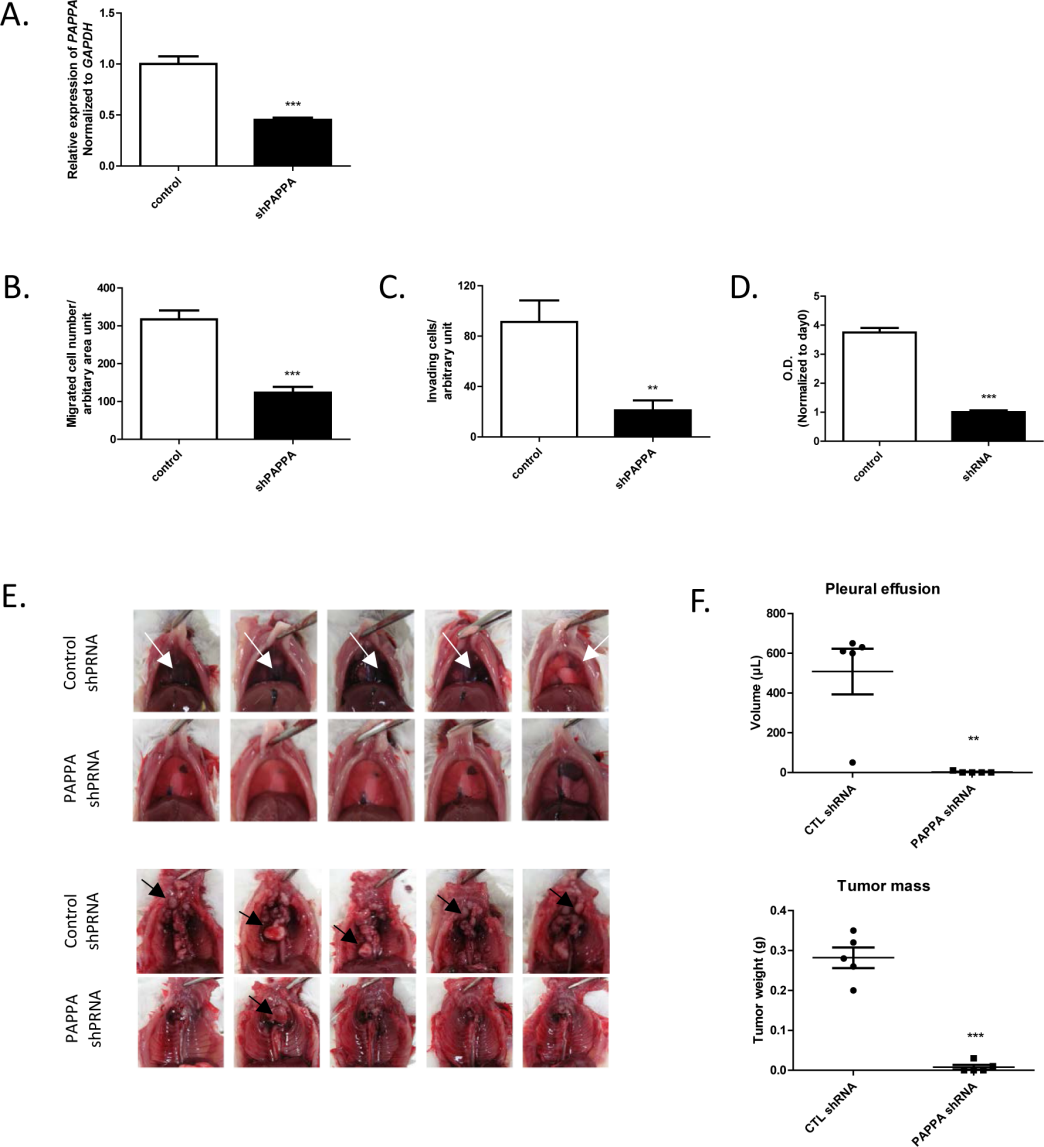
Effects of PAPPA shRNA transfection on migration, invasion, proliferation of NCI-H290 in vitro and tumor growth in orthotopic xenograft model A, After transfection of *PAPPA* shRNA, gene silencing efficacy of *PAPPA* shRNA in NCI-H290 was confirmed by qRT-PCR. *** *P*<0.001, unpaired *t* test. B, The migration ability of NCI-H290 cells transfected with *PAPPA* shRNA was significantly reduced as compared to that of cells transfected with nonsilencing control shRNA. The data are representative of two independent assays carried out in triplicate (means ± SDs, n=3), *** *P*<0.001, unpaired *t* test. C, The cell invasion through the Matrigel was significantly less in *PAPPA* shPAPPA transfected NCI-H290 cells as compared to that in cell transfected with control shRNA. The data are representative of two independent assays carried out in triplicate (means ± SDs, n=3), *** *P*<0.001, unpaired *t* test. D, The proliferation of *PAPPA* shRNA transfected cells was inhibited as compared to that of cells transfected with control shRNA. The data are representative of two independent assays carried out in triplicate (means ± SDs, n=3), *** *P*<0.001, unpaired *t* test. E, The effects of *PAPPA* shRNA on MPM tumor progression in the SCID mouse model of orthotopic xenograft. Pictures of the gross appearance of bloody pleural effusion (upper panel, white arrow) and tumor mass (lower panel, black arrow) of each group were shown. F, The qualitative analysis of pleural effusion (upper panel, ** *P*< 0.01, unpaired *t* test) and tumor mass (lower panel, *** *P* < 0.001, unpaired *t* test).

Our next series of experiments were aimed at further determining the phenotypic effects on MPM cells from *PAPPA* knockdown by establishing stable *PAPPA* knockdown cell lines. To establish stable cell line, we chose MPM cell line NCI-H290, which can be used to establish an orthotopic xenograft model of human MPM. This model reflects the clinical features of MPM patients, such as local tumor progression, malignant bloody pleural effusion in the pleural cavity [3-5, 26]. Silencing of *PAPPA* expression in cells transfected with *PAPPA* shRNA was confirmed (Fig. 6A) before carrying out following assays. Consistent with the results of transient knockdown of *PAPPA* by siRNA, migration ability of *PAPPA* shRNA cells was substantially reduced as compared to that of control shRNA cells (Fig. 6B). We also assessed the invasiveness of *PAPPA* shRNA cells using a Matrigel-coated transwell invasion assay. The decrease of invasiveness of *PAPPA* shRNA cells until 20-30% was even more profound than that of their migratory motility until 30-40% as compared to control shRNA cells (Fig. 6B, Fig. 6C). Also consistent with siRNA experiments, we observed a retardation of proliferation of *PAPPA* shRNA cells *in vitro*, as revealed by MTT assay (Fig. 6D). Despite of the growth retardation observed, the cells seem to be viable for a long time (for at least 14 days we followed) as assessed by morphological observation and Trypan blue exclusion test (data not shown).

Finally, we used a mouse orthotopic xenograft model to investigate the roles of *PAPPA* in MPM tumor progression *in vivo*. NCI-H290 cells stably transduced with *PAPPA* shRNA or control shRNA were orthotopically implanted into the pleural cavity of SCID mice. Mice were sacrificed 21-23 days after tumor cell inoculation. Control shRNA NCI-H290 developed tumors in the pleural cavity in all recipient mice at the time point of sacrifice, and showed classical patterns of diffusely grown pleural-based masses. Four out of five mice developed bloody pleural effusions (hemothorax). On the other hand, *PAPPA* shRNA NCI-H290 only developed traces of tumor mass, and almost no pleural effusions were observed (Fig. 6E, Fig. 6F). Taken together, transfection of *PAPPA* shRNA in MPM cells confirmed the important role of *PAPPA* in cell migration, invasion and proliferation, further revealed its crucial role in MPM tumor development and progression *in vivo*.

## DISCUSSION

In this study, we identified *PAPPA* as a migration-promoting gene in MPM cell lines. By applying a novel approach of combination of transwell migration assay and microarray gene expression profiling, expression level of *PAPPA* is found to be correlated with migration ability of MPM cell lines. The correlation between *PAPPA* expression and migration ability in MPM was further supported by an *in vitro* selection strategy. *PAPPA* expression was found to be parallelly up-regulated in three migratory cell lines as compared to their corresponding parental cell lines. These results strongly suggested that *PAPPA* functionally promotes MPM cell migration.

The role of PAPPA as a local regulator of IGF bioavailability through cleavage of IGFBPs has been well documented [10-12], and the IGF axis was identified as one of the molecular networks involved in the formation, progression and metastatic spread of many cancer types [13], including MPM [14-16, 27]. Based on this observation, we speculated that PAPPA could promote MPM cell migration via IGF-1. To set the stage for the functional role of *PAPPA*, we characterized the major components of the IGF axis in MPM cell lines. IGF-1R and IGFBP1-5 were found to be expressed at varied levels in MPM cell lines. Our further experiments clearly demonstrated that the migration-promoting role can be explained by, at least partly, its proteolytic activity of IGFBP-4 to regulate the bioavailability of IGF-1. Previous studies showed the overexpression of IGF-1 in both MPM cell lines and tissues versus nonmalignant mesothelium at transcriptional level [14, 15], but we did not detected significant expression of IGF-1 at transcriptional level, which was consistent with another large-scale transcriptional profiling study of MPM [28]. The reason for this discrepancy among different reports remains to be determined, which might be explained by the variety of samples investigated. Despite of the discrepancy, given the fact that physiological ranges of serum IGF-1 concentration [29] by far exceed the concentration of IGF-1 needed to have effects on MPM cell migration as demonstrated by us and others [15, 25], we believe that our experiments does have its physiological or even pathogenic relevance. It is worth noting that even though PAPPA did enhance MPM cell migration via IGFBP-4/IGF-1 axis in our experimental setup, we did not exhaust the possibility that nonproteolytic mechanisms might be involved in the migration-promoting function of *PAPPA*.

In the present study, the functional role of *PAPPA* in MPM cell migration was investigated by gene silencing experiments using both siRNA and shRNA. Knockdown of *PAPPA* led to reduced MPM cell migration, invasion and proliferation. Exogenously addition of PAPPA could barely rescue the proliferation of MPM cells from the inhibitory effect of PAPPA silencing *in vitro* (data not shown). This implies that PAPPA might involve in an additional mechanism which has yet to be elucidated. In addition, NCI-H290 cells transfected with PAPPA shRNA, when inoculated into the pleural cavity of SCID mice, failed to develop tumors *in vivo*, while NCI-H290 transfected with control shRNA developed massive tumors and bloody pleural effusions. These observations indicate that *PAPPA* facilitates the progression of MPM and that it might be a potential therapeutic target.

Accumulating evidences support the notion that PAPPA may play a role in cancer. *PAPPA* knockout mice were observed to have reduced incidence of spontaneous cancers [18]. A tumor growth-promoting role of *PAPPA* has been demonstrated in breast cancer [20] ovarian cancer [21] and lung cancer [22] with mouse models very recently. Even though the role of *PAPPA* in cancer has not gathered much attention, IGF-1, the pervasive growth factor whose local bioavailability it regulates, has long been an intense interest for cancer researchers [13]. Actually, several novel therapeutics aimed at the IGF-1/IGF-1R, particularly monoclonal antibodies and small molecule tyrosine kinase inhibitors are under clinical investigation [17]. A phase II clinical trial of cixutumumab, a monoclonal antibody to IGF-IR, which showed encouraging therapeutic efficacy for MPM in preclinical study [23], is currently ongoing for the treatment of patients with mesothelioma. Given the vital physiological function of IGF-1 axis, unforeseen enduring effects on metabolism, body fat, muscle mass, and bone density has been major concerns [17]. In contrast to the pervasive existence and versatile physiological functions of IGF-1 axis, *PAPPA* is barely expressed in adult tissues except placenta, cardiamyocyte and smooth muscle [30], and actually genetic deletion of *PAPPA* extended mean and maximum lifespan of by 30-40% in mice [19]. Therefore, *PAPPA* might serve as a better therapeutic target for MPM with more tumor specificity and less risks of side effects as compared to IGF-1 axis components as targets. Even though most of the studies on *PAPPA* so far suggested its tumor-promoting role is largely IGF-1-dependent, anti-*PAPPA* therapy might provide therapeutic effects beyond the inhibition of the IGF-1/IGF-1R signaling axis. A recent study indicated that IGFBP-4 could inhibit angiogenesis both induced by IGF-1 and FGF-2 [31], Therfore, given the fact that the inhibition of PAPPA can lead to the stabilization of IGFBP-4, targeting *PAPPA* might provide extra therapeutic benefits due to angiogenesis inhibition via IGFBP-4/FGF-2 interaction. In addition, there are evidences showing that PAPPA could promote angiogenesis in the chick chorioallantoic membrane assay via a possible nonproteolytic mechanism [32], further supporting the idea that PAPPA is not simply an alternative target for IGF-1 axis, but a novel target might provide extra therapeutic effects. Actually our preliminary data implied that nonproteolytic mechanisms might be involved in the function of PAPPA in MPM cells (data not shown). Now we are trying to validate these preliminary findings and further investigate the mechanistic role of *PAPPA* in the pathogenesis of MPM.

To conclude, our results supported the idea that *PAPPA* can function as a migration-promoting gene in MPM cells, and revealed that its migration-promoting role could be partially explained by its proteolytic activity. Given the fact that knockdown of *PAPPA* affected cell functions including cell migration *in vitro* and tumor development *in vivo*, we propose that it might be used a novel therapeutic target for the treatment of MPM.

## METHODS

### Cell culture

The human MPM cell lines, MSTO-211H, NCI-H2052, NCI-H2373, NCI-H2452 and NCI-H28, were purchased from the American Type Culture Collection (Manassas, VA). The human MPM cell lines, NCI-H290 and EHMES-1, were kindly provided by Dr. Adi F. Gazdar (University of Texas South-western Medical Center, Dallas, TX) and Dr. Hironobu Hamada (Ehime University, Toon, Japan), respectively. The human MPM cell lines, Y-MESO-14 and Y-MESO-5A, were established as described previously [33]. These MPM cell lines were maintained in RPMI1640 (Nissui Pharmaceutical, Tokyo, Japan) supplemented with 10% heat-inactivated fetal bovine serum (GIBCO, Grand Island, NY), penicillin (100 U/ml), and streptomycin (50 μg/ml). All cell lines were incubated at 37°C in a humidified atmosphere of 5% CO_2_. Cell lines were authenticated by DNA fingerprinting [34].

### Migration and invasion assay

Cell migration was determined by using 24-well transwell chambers with polycarbonate membranes (8.0-μm pore size; BD Biosciences, Bedford, MA). Human MPM cell suspensions in serum free medium were added to the upper chamber at 1 × 10^5^ cells per well. RPMI-1640 culture medium with or without 10% FBS were added in the lower well, and the cells were allowed to migrate for 6 h at 37°C in CO_2_ incubator. Nonmigrant cells were removed from the upper face of the membrane with a cotton swab. The cells were then fixed and stained with Qiff-Quik Staining Kit (Sysmex, Kobe, Japan). The membranes were excised from the chamber and mounted. The migrant cells attached to the lower face of the membrane were counted. For *in vitro* selection of highly migratory cells, we used a method as descripted previously [35]. Briefly, instead of fixing and staining the migrated cells on the lower face of membrane as a routine process, the migrated cells were detached by trypsin treatment, then collected, cultured and propagated. The propagated cells were again subjected to transwell migration assay, and this process was repeated for 3-5 times. Cell Invasion was determined by using Matrigel-coated transwell chambers (BD Biosciences, Bedford, MA) according to protocols similar to that of migration assay except that allowing the cell to migrate for longer time (18 h).

### RNA isolation and quantitative RT-PCR

Total cellular RNAs were isolated using RNeasy Mini Kit (Qiagen, Valencia, CA). After quantification and verification for purity (optical density 260/280 ratios between 1.8 and 2.0) with NanoDrop 1000 (Thermo Scientific, Yokohama, Japan), RNA was reversely transcribed with Takara PCR thermal cycler Dice (Takara Bio, Kyoto, Japan) using High Capacity RNA-to-cDNA Kit (Applied Biosystems, Foster City, CA) following the manufacturer's instruction. Subsequent quantitative RT-PCR was carried out with CFX96 real-time PCR system (Bio-Rad, Hercules, CA) using Applied Biosystems' Taqman gene expression assays for *PAPPA* (Hs01032307_m1), *IGFR1* (Hs00609566_m1), *IGF1* (Hs01547656_m1), *IGFBP1* (Hs00236877_m1), *IGFBP2* (Hs01040719_m1), *IGFBP3* (Hs00365742_g1), *IGFBP4* (Hs01057900_m1), *IGFBP5* (Hs00181213_m1). Samples were normalized to *GAPDH* (Hs99999905_m1) or *ACTB* (Hs99999903_m1) and quantification was determined by using the ΔΔCT method.

### Microarray analysis

Total RNA from MPM cells was isolated using an RNeasy Mini kit (QIAGEN, Valencia, CA). RNA quality was first checked for chemical purity using a NanoDrop spectrophotomer (NanoDrop Technologies, Wilmington, DE) and then assessed for RNA integrity using the Bioanalyzer 2100 (Agilent Technologies, Santa Clara, CA, USA). One hundred ng of total RNA was amplified and labeled using the Affymetrix Whole-Transcript (WT) Sense Target Labeling Protocol, and labeled RNA was hybridized to GeneChip Human Gene 1.0 ST Array (Affymetrix, Santa Clara, CA). Data visualization and analysis was performed using GeneSpring GX (Version 12.1) software.

### ELISA

The human MPM cells (2 × 10^5^) were cultured in medium described above for 24 h, washed with phosphate buffered saline (PBS), 3 ml of fresh culture medium or serum free culture medium were added. After incubation for 24 or 48 h, the culture media were harvested and centrifuged, and the supernatants were stored at −70°C until analysis. Protein levels in supernatants were quantified by ELISA kit for human IGFBP-3, PAPPA (R&D systems, Minneapolis, MN), IGFBP-4 (Abcam, Cambridge, MA) following the manufacturers' instructions.

### Immunoblotting analysis

For the detection of IGFR1 expression in cell lines, the cells were lysed in M-PER (Pierce, Rockford, IL) containing phosphatase and proteinase inhibitor cocktails (Roche, Indianapolis, IN). The concentrations of protein were determined using Bio-Rad Protein Assay Kit (Bio-Rad, Hercules, CA). Blots were incubated with antibodies: IGFR1 (diluted 1:500, 111A9, Cell Signaling, Danvers, MA), β-actin (diluted1:5000, I-19, Santa Cruz Biotechnology, Santa Cruz, CA) overnight. For the detection of IGFBP-4 proteolysis, rhIGF-1, rhIGFBP-4, rhPAPPA and rhtPA (R&D systems, Minneapolis, MN) were incubated at the indicated concentration in 50 mM Tris, 10 mM CaCl_2_, 150 mM NaCl, pH 7.5 buffer for 6 h or overnight at 37°C. Protein mixture was transferred to iBlot™ gel Tranfer Stacker polyvinylidene difluoride (PVDF) membrane (Invitrogen, Carlsbad, CA) according to manufacturer's instruction, the membranes were incubated at room temperature for 2 h with anti-IGFBP-4 (K13RE) antibody (diluted 1:1000, Santa Cruz Biotechnology, Santa Cruz, CA). All analysis were revealed using the horseradish peroxidase(HRP)-labeled anti-rabbit or anti-mouse antibodies (GE Healthcare, Tokyo, Japan), and visualized using SuperSignal West Femto Maximum Sensitivity Substrate (Thermo Scientific, Yokohama, Japan) with a quantitative imaging system LAS4000Mini (Fujifilm, Tokyo, Japan).

### *In vitro* siRNA transfection

Stealth RNAi™ siRNA for *PAPPA* (HSS107591, HSS181743; Invitrogen, Carlsbad, CA) transfection was conducted with Lipofectamine 2000 (Invitrogen, Carlsbad, CA) following the manufacture's protocol. Gene knock down efficacy was confirmed by qRT-PCR at the time points of 24, 48, and 72 h. The resultant cells were used for biological assays at the indicated time point after siRNA transfection. Stealth RNAi™ siRNA Negative Control Med GC Duplex (Invitrogen, Carlsbad, CA) was used as negative control throughout the experiment.

### Lentivirus and infections

MISSION shRNA pLKO.1 constructs (Sigma-Aldrich, St. Louis, MO) were used to make self-inactivating shRNA lentivirus for *PAPPA* [sequence5'-CCG GGG TGA CGG ATG GGA CAT ATT ACT CGA GTA ATA TGT CCC ATC CGT CAC CTT TTT TG-3' (clone NM_002581.3-3690s21c1)], and a non-target random scrambled sequence control (SHC002). For virus transduction, 2 × 10^6^ NCI-H290 cells were incubated with lentivirus plus 5 μg/ml polybrene (CHEMICON International, Temecula, CA) for 24 h. The successfully transduced clones were identified by puromycin (Sigma-Aldrich, St. Louis, MO) selection.

### MTT assay

Cell proliferation was measured by the 3-(4,5-dimethylthiazol-2-yl)-2,5-diphenyltetrazolium bromide (MTT) dye reduction method [26]. Briefly, the MPM cells (2 × 10^3^/well) were seeded in 96-well plates and cultured for 72 h. After incubation, 50 μl of stock MTT solution (2 mg/ml; Sigma, St. Louis, MO) was added to each well, and the cells were then further incubated for 2 h. The media containing MTT solution were removed and the dark blue crystals were dissolved by adding 100 μl of DMSO. Absorbance was measured with a Sunrise Microplate Reader (Tecan Group, Männedorf, Switzerland) at test and reference wavelengths of 550 and 620 nm, respectively.

### Orthotopic xenograft mouse model

Male severe combined immunodeficiency (SCID) mice aged 6-7 weeks (CLEA Japan, Osaka, Japan) were maintained under specific pathogen-free conditions throughout the study. Ethics approval for all animal experiments was obtained from Animal Care and Use Committee of The University of Tokushima. An orthotopic implantation model of human MPM was established as described previously [3-5, 26]. Briefly, 3 × 10^5^ NCI-H290 cells in 100 μl of PBS were injected into the right pleural cavity of mice. The mice were sacrificed 21-23 days after tumor inoculation. The pleural tumors were carefully dissected and weighed; the pleural effusion was harvested using a 1-ml syringe, and the volume of the pleural effusion was measured.

### Statistical analysis

The statistical significances of the differences were analyzed by Student's *t* test, one way ANOVA analysis, where applicable. The correlation efficient and the significance were calculated by Pearson's correlation analysis. The *P*-values less than 0.05 were considered significant in all experiments. Data analysis was carried out using GraphPad Prism version 5.01 (GraphPad Software, La Jolla, CA).

## Supplementary Figures and Table


