# Supplementary material for: Identification of pregnancy-associated plasma protein A as a migration-promoting gene in malignant pleural mesothelioma cells: a potential therapeutic target
Source: Oncotarget. 2013 Jul 8;4(8):1172–84. doi: 10.18632/oncotarget.1126 (PMC3787149; doi:10.18632/oncotarget.1126)
Supplement: Supplementary file 1 [file oncotarget-04-1172-s001.pdf]

Identification of pregnancy-associated plasma protein A as a migration-promoting gene in malignant pleural mesothelioma cells: a potential therapeutic target Huang et al

A.

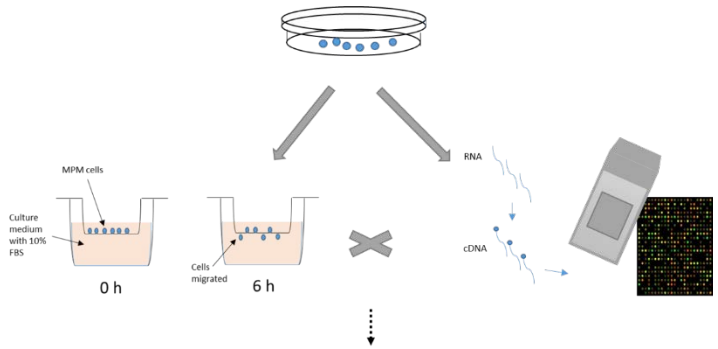

Supplementary Figure 1: Positive correlations between migration ability of MPM cells and their expression levels of candidate genes. A, Schematic representation of candidate migration-related genes identification strategy. Transwell chamber assay was applied to measure the migration ability of MPM cell lines (Figure 1). Gene expression profiles were determined by microarray analysis. Data obtained both from migration assay and microarray was used for analysis to identify candidate genes whose expression levels are correlated with MPM cell migration ability. B, *TNC*, *CGB*, *MAGEA3*, *PAPPA*, *VATL1*, *PTPNC1*, *RYR2* and *PLAT* appeared on the top of the list among the genes whose expression levels are likely to be correlated with MPM cell migration ability**.** X axis: migration ability measured by transwell migration assay; Y axis: gene expression levels determined by microarray. Relative gene expression levels were log-transformed. The correlation coefficients (r) and the significance (*P*) were calculated by Pearson's correlation analysis.

B.

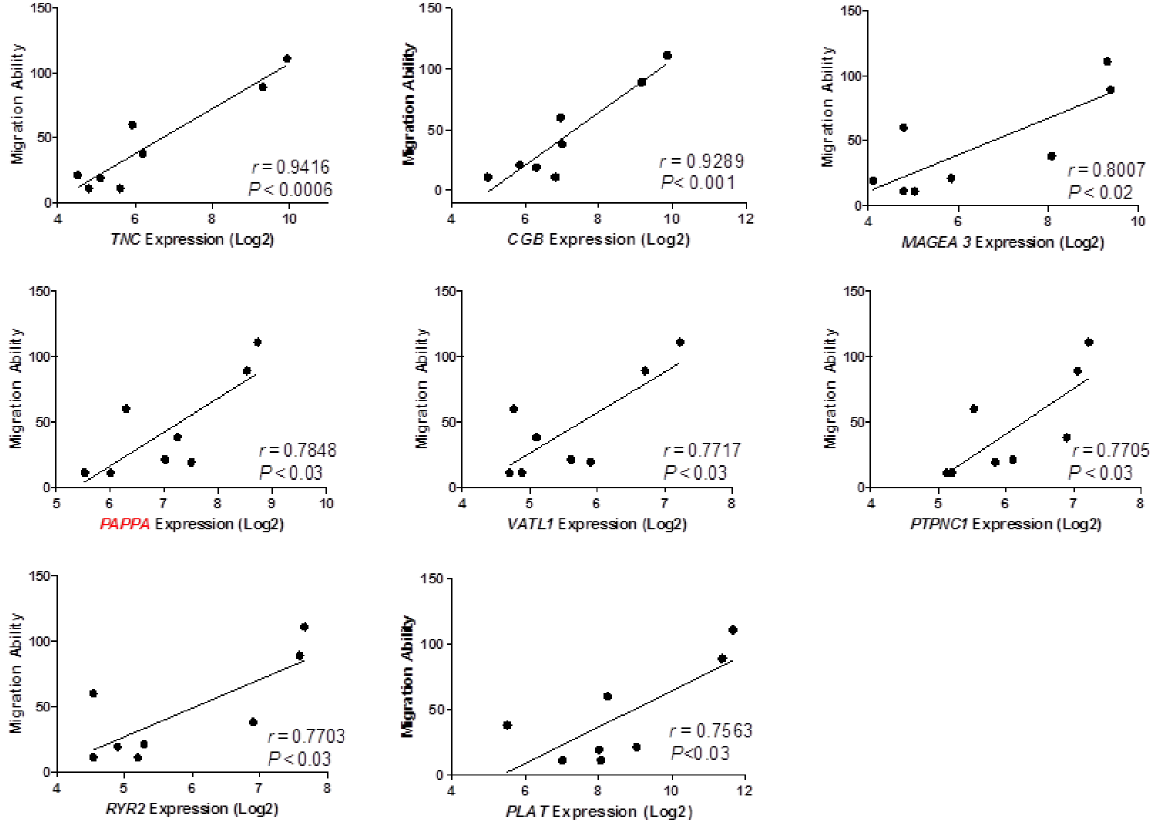

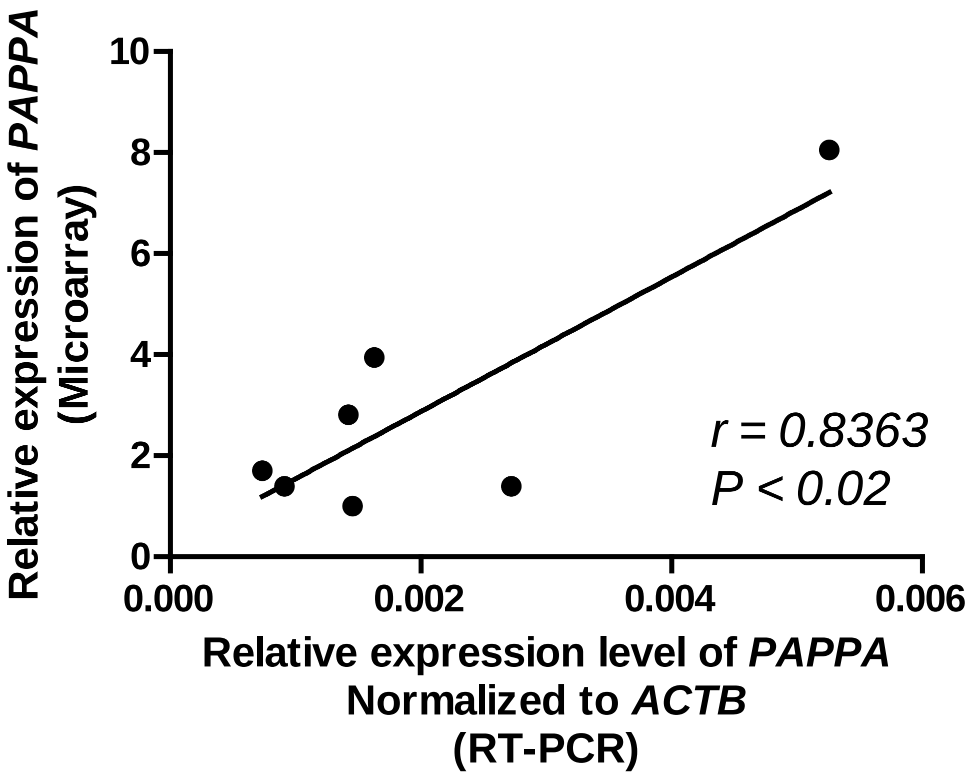

Supplementary Figure 2: Scatter plot of the similarity analysis between microarray and qRT-PCR *PAPPA* expression results. Each plot represents a cell line (MSTO-211H, NCI-H2052, Y-MESO-5A, Y-MESO-14, NCI-H2373, NCI-H2452, and NCI-H290) whose *PAPPA* gene expression level was detected both by microarray and qRT-PCR. Relative *PAPPA* expression determined by microarray was log-transformed, *PAPPA* expression determined by qRT-PCR was normalized to that of *ACTB*. The correlation coefficients (r) and the significance (*P*) were calculated by Pearson's correlation analysis. (* $P<0.05$) (The results of NCI-H28 were excluded from the analysis due to the drastic inconsistence between the data obtained from microarray array and qRT-PCR. The cell line NCI-H28 was also excluded from the following experiments for the same reason.)

Supplementary Table 1.:Relative expression level of defined genes in selected highly migratory MPM cells and their parental cell lines. Genes: *TNC*, *CGB*, *MAGEA3*, *PAPPA*, *VATL1* (*KIAA1576*), *PTPNC1*, *RYR2*, and *PLAT*. MSTO-211H parental cell (211H) versus MSTO-211H highly migratory cells (211HM); NCI-H290 parental cell (H290) versus NCI-H290 highly migratory cells (H290M); Y-MESO-14 parental cells (Y14) versus Y-MESO-14 highly migratory cells (Y14M). Highly migratory cells were selected as described in Materials and Methods. Gene expression profiles were determined by microarray analysis, normalized to that of parental cells, represented in the table as '1".

**Genesymbol**	*KIAA1576*	*PLAT*	*PITPNC1*	*RYR2*	*PAPPA*	*TNC*	*CGB7|CGB5|CGB8|LHB*	*MAGEA3|MAGEA6*
**ID**	10048	25620	11241	1383	26382	27002	14101	28379
**transcript_cluster_id**	7997336	8150509	8009353	7910792	8157487	8163637	8038299	8175747
**211H**	1	1	1	1	1	1	1	1
**211HM**	0.921015	1.131308	1.333958	1.000000	1.704305	1.243317	1.051772	1.000000
**H290**	1	1	1	1	1	1	1	1
**H290M**	1.431530	1.232775	1.131095	1.049902	1.145111	1.563919	1.507479	0.9458078
**Y14**	1	1	1	1	1	1	1	1
**Y14M**	1.324389	1.698860	1.117855	1.682091	1.118571	1.000000	1.000000	1.000000

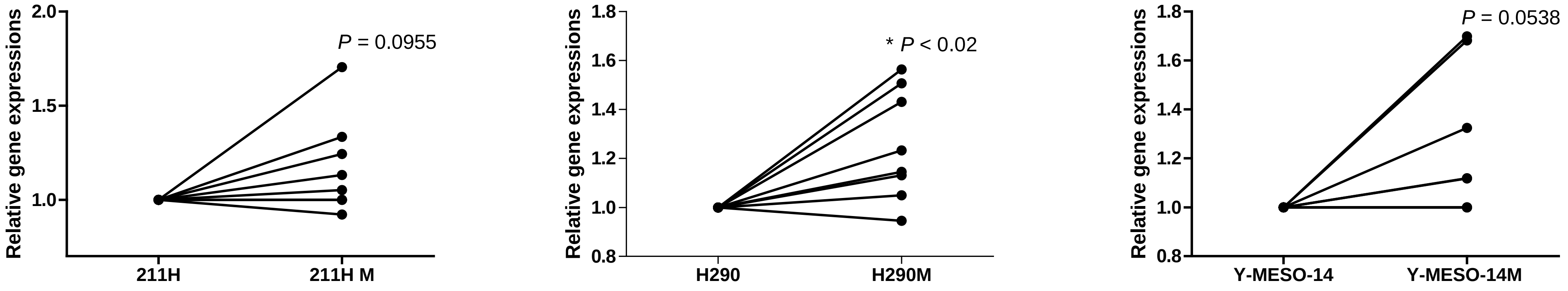

Supplementary Figure 3: Selected highly migratory MPM cells express higher levels of defined genes as compared to their parental cell lines. Each dot represents a defined gene (*TNC*, *CGB*, *MAGEA3*, *PAPPA*, *VATL1* (*KIAA1576*), *PTPNC1*, *RYR2*, and *PLAT*). Gene expression levels were determined by microarray, normalized to that of parental cells. Left, MSTO-211H parental cell (211H) versus MSTO-211H highly migratory cells(211HM); middle, NCI-H290 parental cell (H290) versus NCI-H290 highly migratory cells (H290M); right, Y-MESO-14 parental cells (Y-MESO-14) versus Y-MESO-14 highly migratory cells (Y-MESO-14M). Paired t test. (* P<0.05)
